# Moralizing Consent: Three Field Studies Testing a Student-Led Intervention at University Parties

**DOI:** 10.3390/bs15081025

**Published:** 2025-07-29

**Authors:** Ana P. Gantman, Ajua Duker, Jordan G. Starck, Alex Sanchez, Elizabeth Levy Paluck

**Affiliations:** 1 Department of Psychology, Brooklyn College, Brooklyn, NY 11210, USA; 2 Department of Psychology, CUNY Graduate Center, New York, NY 10016, USA; 3 Department of Psychology, New York University, New York, NY 10012, USA; ajua.duker@nyu.edu; 4 Department of Psychology, Stanford University, Stanford, CA 94305, USA; jostarck@stanford.edu; 5 Department of Psychology, Princeton University, Princeton, NJ 08544, USA; alexsanchez@princeton.edu (A.S.); epaluck@princeton.edu (E.L.P.); 6 School of Public and International Affairs, Princeton University, Princeton, NJ 08544, USA

**Keywords:** moralization, sexual violence, norms, field experiment, consent

## Abstract

Moralization is the process by which preferences become moral values. We investigated a practice that is changing its moral status on college campuses in the United States: affirmative consent to sexual activity. We tested whether messages given to students just before they entered a party impacted their thinking about consent in moral terms—i.e., as a clear issue, with broad consensus, and an imperative to action. At two social clubs on a college campus in 2017, we randomly assigned moralistic vs. informational messages about consent, delivered at the party’s door. At the club that had pre-existing messaging about consent, the moralistic (vs. informational) message increased students’ thinking about consent in moral terms. By contrast, in the club without prior consent messaging, the informational (vs. moralistic) pledge increased students’ thinking about consent in moral terms. We then investigated and found weak evidence for a small reduction in administrative-level student conduct complaints compared to prior and subsequent years as a result of a one-night consent message treatment unique to each of the 12 clubs hosting a party. Theoretically, our findings make progress toward understanding processes of moralization. Pragmatically, they suggest the importance of locally tailored messages that reflect and shape the values of social groups.

## 1. Moralizing Consent: Field Experiments Testing a Student-Led Intervention at University Parties

Moralization is the process by which preferences become values (Rozin, 1999; Rozin et al., 1997; Rozin & Singh, 1999), or ideas increase in their degree of relevance to a person’s sense of right and wrong (Rhee et al., 2019). Moralization has been studied primarily in two forms: moral recognition and moral amplification (Rhee et al., 2019). Moral recognition occurs when an idea or behavior not previously thought to be relevant to the moral domain shifts into moral relevance. For example, a person who experiences moral recognition may have previously thought that smoking cigarettes is a matter of personal preference but now thinks of smoking as morally wrong. Moral amplification occurs when an idea or behavior previously thought to be relevant to a person’s moral values becomes more central to their understanding of right and wrong. For example, a person who experiences moral amplification may have previously thought that smoking was somewhat morally wrong but now thinks of smoking as extremely wrong and conflicting with their core belief in not harming others. As of now, there is relatively little theorizing or evidence on the conditions that trigger or facilitate moralization, especially the initial process of moral recognition (Rhee et al., 2019). The goal of this paper is to evaluate a real-world intervention aimed at moralizing an idea for individuals and institutions that may not recognize or only somewhat recognize its moral relevance. That is, the current paper begins to fill this empirical gap, starting with understanding the role of institutional messaging. Here, we test the idea that when institutions issue moralistic messages, or messages that make direct claims about the positive moral status of an issue, the messages may persuade individuals to think about an idea in moral terms—but only to the extent that the institution has already publicly recognized the issue as a moral one. Evidence of the impact of these types of interventions can move our field closer to an overarching theory of how ideas and behaviors become moralized. In this case, we examine the idea and practice of sexual consent, specifically on college campuses.

## 2. What Is the Moral Status of Consent on College Campuses in the United States?

Consent to sexual activity is often defined in terms of its relationship to sexual assault. For example: “[s]exual assault is broadly defined as any sexual encounter absent of clear, uncoerced, affirmative consent given or obtained by a sexual partner” (Ortiz & Shafer, 2018). While college students likely agree that rape and sexual assault are a moral issue (Edwards et al., 2014; Koss et al., 1998), victims are often blamed for assault (see, e.g., Jordan & Sommers, 2024; Niemi & Young, 2016), and it does not follow that all students view obtaining and maintaining consent in the context of sexual activity as a moral obligation. While it may seem theoretically that the positive moralization of consent follows from moral opposition to assault, this is not the case in practice. Among college students (even those within the same university context), definitions of consent, understandings of the kinds of verbal and nonverbal behavior that constitute consent, and which sexual encounters count as clear cases of sexual assault, vary widely (Baldwin-White, 2021; Goodcase et al., 2021; Hills et al., 2021; Marcantonio & Jozkowski, 2023; Pella & McClung, 2024). Conversations about the meaning of consent, especially on college campuses, are complex, fraught, and ongoing (Bednarchik et al., 2022; Hirsch et al., 2019; Muehlenhard et al., 2016; Shafer et al., 2018). As one illustrative data point, college students report that male students do, and are expected to, use coercion to obtain consent (Jozkowski & Peterson, 2013; McKinnon et al., 2025; Oswald & Russell, 2006), but not all incidents that involve coercion are labeled as instantiations of sexual assault (Muehlenhard et al., 2016).

Universities aim to educate students about affirmative consent and to convince students of their moral obligation to avoid and to protect others from non-consensual sexual activity (e.g., itsonus.org/about/; endrapeoncampus.org/about/; ctureofrespect.org/about-us/; Ortiz & Shafer, 2018). Unfortunately, sometimes these efforts are one-time or inadequate (Freitas, 2018; Htun et al., 2022; Lee & Wong, 2024; Richards et al., 2023) and are undercut by incidents on campus in which some students deride consent (Gajanan, 2018; Stack, 2016b) and by the actions of national institutions like the recant of the Obama administration “Dear Colleague” letter urging universities to take sexual assault cases seriously (Saul & Taylor, 2017). Less explicitly, different events on campus, such as Division 1 football games, are reliably associated with increases in daily reports of rape (Lindo et al., 2018). University students are likely arrayed on a spectrum in terms of how moralized their attitudes about consent are (Hirsch et al., 2019; Li et al., 2024; Muehlenhard et al., 2016; Rhee et al., 2019; Shafer et al., 2018; Wylie et al., 2022); it is likely that different spaces on campus—such as the women’s center, a fraternity, or the student newspaper—are home to group identities, practices, and expectations that differentially reinforce how moralized the issue of consent may be for individuals in those groups.

## 3. What Leads to Moralization?

How do some students, embedded in local social institutions like social clubs and sports teams, come to moralize consent? So far, researchers have investigated the causal role of incidental (or independently activated) emotions with mixed results (and never about sexual consent). A meta-analysis of 13 studies investigated whether experiencing the feeling of disgust, induced independently from the target of the moral judgment itself, led to a change in moralization such that people changed from thinking an issue is not moralized at all to one that is in the moral domain. It seems that emotions are more likely to affect the degree to which an issue is moralized than a category change from “not-moral” to “moralized”. Specifically, when people see graphic depictions of abortion (but not when they experience disgust activated by irrelevant means), they are more likely to endorse increased intensity in their moral conviction about abortion, an effect that is mediated by their feelings of disgust (Skitka et al., 2017). Messages that seek to moralize ideas for persuasive purposes (Chen et al., 2009; Van Zomeren, 2013) can also lead to moral amplification. Moralized messages are particularly persuasive when the audience has already recognized the issue as a moral one (Luttrell et al., 2019) and when they are tailored to the audience’s specific values (Feinberg & Willer, 2019). For example, in the U.S., political conservatism is positively related to patriotism, and when messages arguing for protection of the environment—sometimes negatively correlated with conservatism—are framed as patriotic, this can increase support for environmental protection among U.S. conservatives (Feinberg & Willer, 2019; Feygina et al., 2010).

An unanswered question in this literature is how institutions might instigate moralization for heterogeneous groups. For example, in a country as heterogeneous as the U.S., Supreme Court rulings on morally relevant topics like abortion have been shown to affect Americans’ impressions of whether other Americans support legalized access to abortion (Clark et al., 2024). In the case of abortion, the Court’s ruling did not affect Americans’ moral convictions about abortion—not for Republicans, who on average hold moral convictions against abortion, and not for Democrats, who on average believe in a moral case for it (Clark et al., 2024). However, sometimes, judgments about which attitudes are common can be used as a heuristic for what is moral (Lindström et al., 2018). This suggests that the role of institutional signals in individual- or societal-level moralization is an open empirical question. The answer is likely to depend on contextual features of both the institutions that send moral messages and the people intended to receive them. For example, it is possible that institutions that are more proximate to groups, like a town’s political organization or a student group, may be more powerful purveyors of moral messages than more distal institutions like the Supreme Court. However, we do not currently know much about whether institutional signals can affect moralization at the individual level.

## 4. How Do We Measure Individual Moralization?

One challenge in seeking to better understand the processes involved in moralization is measurement. Ideas that are changing in moral status in society may be sensitive or subject to significant experimenter demand, and so it is useful to measure changes in moralization without asking participants directly. Ideally, the measurement tools must be valid at different levels of moralization, ranging from ideas that are consistently highly moralized, like murder, to those that may or may not be moralized at all, like getting a divorce. In addition, it is useful if measures of moralization are correlated with, but not necessarily identical to, highly face-valid measures of moral values and moral convictions (Skitka et al., 2005). For instance, a highly face-valid measure of whether someone has moralized attitudes about sexual consent might read, “How much are your feelings about sexual consent connected to your core moral beliefs or convictions?” And if the person posing the question is a friend who is a feminist activist or a friend accused of misconduct, they might feel pressure to answer in one way or another.

How can we know when an idea is changing in moral status? One way to measure whether (and to what extent) an individual has moralized an idea or not, without asking them directly, is to search for a “signature” of a moralized idea. Recent research has converged on and clarified the signature of a moral idea (Rhee et al., 2019; Wylie et al., 2022). Specifically, people are relatively more likely to understand their own moral judgments as objective (Goodwin & Darley, 2008; Heiphetz & Young, 2016), as a universal standard for others (Van Bavel et al., 2012), as widely shared (Lindström et al., 2018), and as requiring individual (Skitka, 2014) and collective action (Van Zomeren, 2013). We can also test for evidence that an idea has become moralized at the societal level. For example, behaviors in line with moral values are more likely to receive institutional funding, as well as validation in the form of laws or taxes (Rhee et al., 2019; Rozin, 1999; Rozin et al., 1997; Wylie et al., 2022).

Thus, measures of an idea’s perceived objectivity, universality, social consensus, and imperative to action can illuminate whether an idea has been moralized. These features of moralized ideas at both the individual and societal level are not the ideas themselves (e.g., smoking is wrong), but they can serve as indicators that suggest a given idea falls within the moral domain of an individual or group. For example, at the individual level, people who condemn smoking cigarettes are also very likely to say that the wrongness of smoking is objectively true and unambiguous (whether it is or not), or “black and white,” (Wylie et al., 2022) and at the institutional level, some U.S. states, like New York, place a relatively high sales tax on cigarettes to discourage smoking (American Lung Association, 2024; Rozin & Singh, 1999).

We tested the effects of two types of institutional messages about sexual consent (moralistic vs. informational) within small local institutions on a college campus that have and have not publicly recognized the issue of consent as a moral good. Consider two groups on a college campus: one that has explicitly signaled its commitment to sexual consent as a moral issue with statements, signage, or other practices, and another that has been silent on the issue. A moralistic message about consent will likely be heard differently by members of the first club compared to members of the second. Our research asks the following questions: Do moralistic or informational institutional messages about sexual consent trigger more moralized thinking? Is the effect of the message bounded by the institution it comes from?

The vast majority of existing work on moralization processes concerns smoking and meat eating (Feinberg et al., 2019; Rhee et al., 2019; Rozin, 1999) and some on attitudes towards abortion (Skitka et al., 2017). As discussed in public and within the research, the moralization of all of these issues is negatively valenced. By this, we mean that negative emotions (i.e., disgust, outrage) are associated with the moralized attitudes and judgments ascribed to these issues (e.g., Skitka et al., 2017), and that such issues are thought of as wrong, immoral, and/or unethical, prompting moral opposition (Wylie et al., 2022). Here, we focus on the moralization of sexual consent. Instead of testing moral opposition to sexual violence, we test moralistic messages aimed at the positive moralization of consent, which emphasize sexual consent as an imperative, universal, and normative aspect of sexual activity. Institutions, particularly universities, are expending great effort and resources to regulate and increase sexual consent practices on college campuses through both policy and on-site education efforts that seek to change how people think about consent and sexual violence (Porat et al., 2024).

## 5. Study Context: Consent and Sexual Assault

At the university where we collected our data, a Title IX survey found that the rate of sexual assault among undergraduates was similar to national rates: 20% of undergraduate women reported experiencing sexual misconduct in a given year. Of these women, many (38%) first encountered their assailant at one of eleven student-led social clubs on campus, where most parties are held. These social clubs are independent, historically male but currently co-ed, and secular institutions without formal ties to the university. They represent the most popular dining and social option for upperclassmen. They are located in houses along the same street on campus and, notably, vary (among other things) in terms of whether and how they raise and address the issue of consent with their members and non-member party attendees.

At the time that we began data collection (Spring 2017) the #MeToo movement was just emerging into mainstream consciousness, though the issue of sexual assault was already salient on college campuses due to both recent controversies (Erdely, 2014; Nathanson, 2014; Stack, 2016a) and longstanding feminist activism on campuses (e.g., Brownmiller, 2000; Hirsch & Khan, 2020). In 2017, the student leaders of one social club on campus instituted a new consent practice that was public, mandatory, approved by the student membership of the club, and developed independently of the university administration (and in response to a high-profile sexual assault at a peer institution; Stack, 2016a). At the club’s weekly late-night parties, open to all university students, a student wishing to enter the party would be required to read aloud a definition of consent. Students referred to this new consent practice as a “pledge”, though there was no action promised or associated with it. Rather, the pledge was a concrete and informational definition of consent (see Section 7.1.2 in Methods for full wording). The student leaders of the club labeled the consent practice as a pledge, which echoed a university-wide practice that requires all students to write and sign a pledge attesting that their work was not plagiarized before turning in completed exams and papers for formal grades.

We theorized that the existence of a club’s public, mandatory, student-initiated group practice would suggest to students that consent was moralized by the members of this social club. Because students (even those within the same institution or university) vary in the extent to which they think of consent in moral terms (Hills et al., 2021), and we did not collect data that assessed their pre-treatment moralization of the issue, we contend that both processes of moral recognition and moral amplification might occur, depending on students’ previous attitudes. We then asked the following question: What kinds of messages encourage moralization among people visiting this social club, when it has already signaled its ideas regarding consent?

We met with student leaders of the social club, who expressed interest in testing new language in their pledge. As researchers, we asked the following: Would students attending parties at this club respond to a message directly moralizing consent (as compared to the current informational message) by thinking about consent in more moral terms? In other words: would a message directly moralizing consent (as compared to the current informational message) amplify the moral relevance of consent to the group? The club agreed to randomize which type of pledge individuals saw at the door: either the existing informational pledge or a new “moralistic” pledge. The moralistic pledge defined engaging in sexual activity without obtaining consent as violence, portrayed consent as a widely agreed-upon imperative, and used morally relevant, often salient language (Gantman & Van Bavel, 2014, 2015). To assess the effect of reading either pledge, we measured the extent to which students thought about consent in moral terms at the end of the party.

While we were conducting our experiment with the first social club, another club invited us to replicate our experiment at one of their parties. This club had no practice comparable to the first club’s pledge that would suggest to students that it moralized consent—an ideal comparison context. Finally, we sought to investigate campus-wide rather than club-level effects. To do this, we evaluated the effect of a consent pledge on disciplinary behavior following a campus-wide pledge night. On the night following the end of each semester’s exam period, all of the social clubs open their doors to guests for parties. This single night afforded us a unique opportunity to look for campus-wide effects of such a pledge on subsequent behavior, allowing us to search for behavioral evidence that consent messages not only changed how students thought about consent, but also how they behaved (see Porat et al., 2024 on the need for measurement practices of this kind). This is a very different campus-wide intervention than university-mandated training about consent and sexual assault in several key ways. First, it functioned as the continuation of a student-led initiative (i.e., a “bottom-up” institutional intervention). Second, each club chose its own message, which means the messages were unlikely to be out of step with previous club messaging (or lack thereof) about consent. Third, the intervention occurred in the place and time that sexual assault is highly likely to transpire within this campus setting (i.e., at campus parties themselves, rather than as part of educational seminars, which typically take place during the day in classrooms, administrative buildings, or online). Together, the three studies address questions about how locally tailored institutional messages affect individuals’ responses to moralistic messages during a period marked by a notable peak in public discourse around consent and sexual assault.

All studies were conducted in compliance with Princeton’s Institutional Review Board. Our IRB determined that a written consent procedure would contain the most identifying information of the whole procedure. For all studies, we opted for oral consent, recording minimally identifying data (self-reported year and gender). However, since many students attend parties at the social clubs, we can approximate the demographic composition of the party attendees based on university-level data. Students typically arrive at this university immediately after or one year after completing high school, and so the average age of a full-time student at this university is approximately 20. The average age of a first-year student is approximately 18. At the time we ran this study, 42.6% of students were White non-Hispanic, 21.4% Asian, 10.1% Hispanic/Latino, 7.7% Black or African American, 4.3% two or more races, 1.5% unknown, and 0.3% Native American or Pacific Islander. A total of 12% of the students were from outside the U.S., and 60% of students were on need-based financial aid.

Finally, across all studies, we surveyed students as they exited a party on their college campus. Some students had consumed alcohol. As the legal drinking age in the country of the site was 21 years old, we did not record any information about alcohol consumed, as we did not want a written record of any illegal behavior or for students to fear that we were collecting this information about them. All surveys were conducted on paper and were coherently and legibly answered. We consider the presence of alcohol at the study site to be a strength of the study rather than a flaw. Alcohol co-occurs with sexual assault on college campuses and elsewhere (for a review, see Gantman & Paluck, 2021). For any intervention to successfully change the moral status of consent in the minds of college students, it would need to be effective in exactly this kind of setting. Contrast this outcome measurement with survey evidence that students sitting in a class or at breakfast regard consent as a moral issue. While still informative, these more formal and sober settings may not sufficiently capture students’ views on consent in moments when it is most relevant.

### 5.1. Hypotheses

How do institutional processes launch or amplify ideas in the moral domain? We tested specifically whether students attending parties at social clubs using a consent pledge would moralize consent to a greater extent after exposure to a moralistic (vs. informational) pledge about consent at the party’s entrance. Our hypothesis about which message would lead to greater moralized thinking about consent differed across study sites (i.e., social clubs on campus). We collected data at two different social clubs (on different nights). One social club had already instituted the informational pledge. The second one had not.

At Club 1, where the consent pledge was initiated, people attending parties at this club had already been exposed to the informational consent pledge, and knew it was created by its members and leadership. This was likely perceived as a signal that the club leaders cared about consent already. Thus, we predicted that the moralistic (vs. informational) message would cause more moralized thinking about consent (evidence, perhaps, of moral amplification). At Club 2, there were no pre-existing consent practices, so no indication either way about whether the group might already moralize consent, but student leaders from this club were involved in administering both the moralistic and informational consent messages on the night of data collection. Thus, we predicted that moralistic (vs. informational) messaging might cause either greater or less moralization of consent. We pre-registered this bidirectional prediction at Club 2 because, unlike at Club 1, we had no prior indication of how the consent pledge practice would be received, as it had never been done before in this setting.

We conducted post hoc sensitivity power analyses using G*Power software ver. 3.1.9.6. (Faul et al., 2007) given that the size of the population at each party was uncertain in advance. With a two-tailed alpha significance criterion of 0.05 and a power criterion of 80%, we were able to detect small to medium effect sizes (f2 from 0.045 to 0.079) in Experiment 1 and small effect sizes (f2 from 0.025 to 0.041) in Experiment 2. This range is representative of the small average effect size observed in a recent meta-analysis of RCTs of interventions on attitudes regarding sexual harassment and assault (Porat et al., 2024).

In Study 3, we sought to test whether a campus-wide consent intervention could have a measurable impact on actual student behavior. We selected an evening of campus-wide festivities in 2018, and asked all student clubs to design and administer their own consent pledge, in the hopes of observing changes in how often students face disciplinary action related to issues of sexual misconduct and disrespectful party behavior more generally. We predicted that when we allowed all clubs on campus to design and administer their own consent pledge on a night of campus-wide celebration, the pledge would lead to decreases in student disciplinary infractions (compared to the same celebration period in other academic years). This prediction was based on the expectation that the content of at least some of the pledges would result in increased moralization of consent (i.e., the Club 1 hypothesis) and that the widespread uptake of pledges across all organizations for the night would establish a strong norm that students and student institutions supported consent at the university. We expected that both factors could lead to a reduction in untoward behavior measured by campus disciplinary records.

### 5.2. Transparency and Openness

For Studies 1 and 2, we pre-registered all hypotheses before data analysis, manipulations, measures, and analyses; pre-registrations, materials, code, supplement, and data can be found at https://osf.io/w7btc/.^1^ The analyses for Study 3 are included in the OSF page. We cannot make the university raw data publicly available per our agreement with the university on the use of sensitive administrative records of student disciplinary infractions.

## 6. Measuring Moralized Ideas

When conducting our experiments in the field, we were wary of using dependent measures that would cause reactance or clue participants into the main questions we were studying. As college students, the participants in our experiments are well-versed in university-approved responses regarding sexual assault and consent given their compulsory engagement in required online and in-person training sessions they receive on sexual assault, consent, stalking, and intimate partner violence. This vulnerability to experimenter demand is another reason why we chose to measure moralization according to its theorized “signature”, as discussed above. We used four items that correlate with more face-valid measures of moral judgments like “It is morally wrong to [X]” (Wylie et al., 2022) and adapted them to apply to consent and to the pledge. Specifically, we measured perceived objectivity or whether the issue was perceived to be “black and white” rather than nuanced (Goodwin & Darley, 2008; Heiphetz & Young, 2016) by asking “Is consent confusing?” (reverse-scored); perceived universality (Van Bavel et al., 2012) by asking “Should everyone pledge?”; and normativity (Lindström et al., 2018) by asking “what percent of your friends appreciate the pledge?” To ask about whether the issue mandated action (Skitka, 2014), we asked “How responsible are you for preventing sexual assault?”.

To validate our dependent measures, we ran an additional pre-registered study on Amazon’s Mechanical Turk (N = 160; https://osf.io/w7btc/) to test to what extent our items were correlated with two face-valid measures of moral judgments. The two face-valid moral items were “To what extent is sexual consent a matter of fundamental right and wrong?” and “How much are your feelings about consent connected to your core moral beliefs?” (Muehlenhard et al., 2016). We found significant correlations between the face-valid items and all four of our field survey items (all at *p* < 0.010), suggesting that our measurement of a “moral signature” of consent does suitably measure the extent to which participants moralize consent.

In addition to consent, we re-posed our survey items with two additional moral topics—fidelity in marriage and online dating—that we expected to be high and low in moralization, respectively. Alphas for the three composite scales measuring moralization of infidelity, consent, and online data were all above 0.7 (we pre-registered an expectation that they would all have an alpha of above 0.6 to indicate a reliable scale). As expected, we found that infidelity in marriage was moralized on average more than online dating (on a 0 to 4 scale;^2^ M marital infidelity = 3.09, SD = 0.77; M online dating = 1.99, SD = 0.72). Consent was moralized significantly more than online dating, no differently than fidelity in marriage (M = 3.16, SD = 0.73). This both validated our more subtle measure of moralized thinking against frequently used face-valid items, and confirmed our assumption that sexual consent is a topic that is less unilaterally moralized (or not) compared to other similar topics.

## 7. Study 1

Study 1 was conducted in Spring 2017 at a large party at Club 1, which previously mandated party attendees to read aloud a pledge about consent at the door. We report how we determined our sample size, all data exclusions (if any), all manipulations, and all measures in the study (Simonsohn et al., 2012).

### 7.1. Method

#### 7.1.1. Participants and Procedure

All undergraduates who entered Club 1’s party on the night of the experiment were required by the club to read one of two randomly assigned messages about consent before entering the party: either the moralistic or the informational pledge. We sought to survey all party-goers as they left the party (N ≈ 300), 176 (64 males) of whom completed our survey, and 161 of whom indicated their year (56 freshmen, 63 sophomores, 22 juniors, and 20 seniors). There was no failure to treat—all students read the pledge at the entrance, as required by the club. When they exited the club, students could opt-in to our survey for a slice of pizza and a bottle of water in return, offered from a booth by our research team. Participants completed surveys on their own using a clipboard. Studies 1 and 2 were conducted in accordance with the Declaration of Helsinki, and approved by Princeton Institutional Review Board, Protocol #7900. The IRB approved a procedure in which participants were not required to provide their written consent due to the lack of personally identifying information collected.

Randomization to moralistic or informational pledge: At all of the social clubs on the campus, students first form a line at the door to show a security guard their identification. During our experiment, one of our research assistants waited for them to complete this (separate) stage and then directed each student (following a random number list) to one of two lines. In one line, participants received the informational pledge, and in the other, the moralistic pledge. Students in both lines followed the same procedure—when they reached the front of the line, a student representative of the club, wearing a university sweatshirt, held up a paper with the pledge printed in large font. Students were asked to read the pledge aloud, and then received a stamp on their hand (another normal procedure for many clubs). Specific to our study, the stamp was unique to each pledge so that the research team could identify which pledge the student read when they filled out their survey before exiting the party.

#### 7.1.2. Materials

Informational vs. moralistic consent pledge: The informational pledge, which was written and previously implemented by the student leaders of Club 1, read as follows: “Consent is asking for and receiving affirmation from someone of sound mind before and while engaging in their personal space or belongings, and can be revoked at any time.” The moralistic pledge, written by our research team and approved by student leaders at Club 1 read: “[university name] students know: for sexual activity to be right, it must be consensual. I stand with my fellow [university name] students: Nonconsensual sex is not sex; it’s violence. I am responsible for getting and receiving consent, which cannot be given when incapacitated and can be revoked at any time.” The moralistic pledge evokes multiple psychological constructs strongly associated with moralization processes (Wylie et al., 2022), including identity, morality, and personal responsibility. We intentionally created the strongest possible manipulation to maximize the possibility that it would resonate with students throughout and after the party. We refer to it as the moralistic pledge for brevity.

Moralizing consent: The survey consisted of 12 items. The first question asked about perceived social norms regarding the consent pledge: “What percent of your friends appreciate the pledge?” Next, we assessed whether participants preferred universal adoption of the pledge: “Should everyone at [university name] read the pledge?” We assessed clarity of consent by asking “Is consent confusing?” and how responsible students feel for preventing sexual assault. All items used Likert scales from −3 (“strongly disagree”) to 3 (“strongly agree”). Participants who thought about consent in moral terms to a greater extent were expected to respond with higher ratings to these questions, except for the clarity of consent question, which was reverse-scored for analyses.

Three questions were included to assess whether the different pledge types affected students’ overall party experience and whether they changed students’ feelings of safety at the party (we did not make predictions regarding these items). We asked (1) how much they enjoyed the party, (2) how safe they felt at the party, and (3) whether or not they would bring their little sister to the party. For exploratory purposes, we noted the approximate time participants were surveyed; these data did not meaningfully and consistently predict any outcomes. Finally, we asked about participants’ recall of the words in the pledge, identification with the university, and one question of interest to the social club, all of which are not pertinent to the current analyses (see OSF for the complete survey).

### 7.2. Results

**Analytic approach:** In an unpreregistered pilot study, we found that all of our dependent variables exhibited extreme skew that favored positive responses to the consent pledges. For example, for the question “Should everyone at [university name] read the pledge?”, where agreement was measured on a scale from −3 (“strongly disagree”) to 3 (“strongly agree”), the skew was −1.9, and the median was 3. Following our pre-registered code, we separated treatment assignment data from the dataset and re-coded every answer to indicate strong agreement or less than strong agreement: scores from −3 to 2 were re-coded as 0, and scores of 3 re-coded as 1. Similarly, for our norms question about the percent of friends who appreciated the pledge, we re-coded all answers from 0 to 99% as 0 and all answers of 100% (and a few above) as 1. We also observed a high proportion of first-year students in our sample, and thus treated class year as dichotomous, contrasting freshmen (35% of the sample) with sophomores, juniors, and seniors. Then, we re-merged the data with the treatment variable.

In our pre-registered linear regressions, we first examined the bivariate effect of treatment—the moralistic pledge vs. the informational pledge—on each transformed dependent variable. We then added gender and school year (dichotomized) as covariates and ran a third model interacting treatment with covariates. We calculated robust standard errors for all regression coefficients and repeated all analyses with logit models, given the dichotomous outcomes. Linear models, which did not differ from logit models, are presented for ease of interpretation. Our survey results, with the simple and covariate-adjusted OLS models, are summarized in Table 1. The fully interacted models, which reach the same conclusions, are reported in the Supplementary Materials available on OSF (https://osf.io/w7btc/).

**Moralizing consent**: Overall, we found some support for the hypothesis that, in Club 1, a moralistic message about consent would lead to greater thinking about consent in moral terms (see Table 1). Specifically, in the bivariate and adjusted regressions, we found an effect of the treatment on whether participants found consent to be confusing, B = −0.22, SE = 0.07, 95% CI [−0.36, −0.08], *p* = 0.002, such that participants who received the moralistic pledge were less likely to express any confusion about consent. We also found that students who read the moralistic pledge were more likely to find themselves extremely responsible for preventing sexual assault, B = 0.17, SE = 0.08, 95% CI [0.02, 0.32], *p* = 0.028. We did not find an effect of the moralistic pledge on participants’ judgment of whether everyone should read the pledge, B = 0.08, SE = 0.07, 95% CI [−0.07, 0.22], *p* = 0.291, or of whether all of their friends appreciate the consent pledge, B = 0.16, SE = 0.09, 95% CI [−0.03, 0.34], *p* = 0.102 (see Table 1).

**Party experience:** Overall, our treatment did not affect party experience in terms of who felt safer, *p* = 0.825, of whether they were likely to bring their little sister to the party, *p* = 0.332, and of their enjoyment of the party, *p* = 0.829.

In sum, at Club 1, we found some evidence that the moralistic pledge increased thinking about consent in moral terms compared to the informational pledge.

## 8. Study 2

We replicated our experiment in a new club during the same time period (Spring 2017), where there were no prior consent awareness practices. Accordingly, we sought to learn more about how individuals would respond to the moralistic pledge in an environment with a relative absence of institutional signaling about consent.

### 8.1. Method

#### 8.1.1. Procedure

The procedure from Study 1 was replicated nearly identically, with a few exceptions, which we note. Following the request of Club 2’s leadership, students attending the party could opt out of reading the pledge, and were still allowed to attend the party. If participants did this, they did not receive a stamp on their hand and were not surveyed at exit. We also included a question on the survey about whether people had taken the survey before at Club 1. Too few people answered this question, which was easy to miss, as it appeared in the top right corner of the paper survey, for analysis.

#### 8.1.2. Participants and Design

Of the 538 total party attendees, 12 refused the moralistic pledge, 2 refused the informational pledge, and 5 entered the party before being assigned a condition (19 total). A total of 328 students (167 male) completed the survey after exiting the party, 305 of whom indicated their year (102 freshmen, 59 sophomores, 60 juniors, and 84 seniors). We attempted to measure whether students in Study 2 had previously taken the survey in Study 1, but too few students answered this survey question for the research team to conduct meaningful analyses inclusive of this measure.

#### 8.1.3. Materials

Study 2 materials were identical to those used in Study 1, except for the following addition: we assessed the degree to which participants felt that sexual assault was a problem on campus, using the same Likert scales (from −3, “strongly disagree”, to 3, “strongly agree”).

### 8.2. Results

**Analytic approach:** We used the same analytic approach as in Study 1 by examining raw data without treatment assignment and pre-registering analyses after transforming skewed data. Responses to our new question, “Is sexual assault a problem at [university name]?”, were not positively skewed, so we did not re-code answers. No cases were excluded from analysis. Table 2 summarizes our survey results. Logit models returned the same patterns of results; fully interacted models are reported in the Supplementary Materials.

**Moralizing consent:** In contrast to Study 1, at Club 2, we found evidence suggesting that exposure to the moralistic pledge led to thinking about consent in moral terms to a lesser extent, compared to the informational pledge (see Table 2). Specifically, participants who read the moralistic pledge were significantly less likely than those who read the informational pledge to believe that everyone should pledge, B = −0.18, SE = 0.05, 95% CI [−0.29, −0.07], *p* = 0.001. We found a modest effect of the moralistic pledge on whether participants found consent to be confusing, B = 0.11, SE = 0.05, 95% CI [−0.002, 0.21], *p* = 0.054. We found an indeterminate effect of the moralistic (vs. informational) pledge on participants’ estimates that all of their friends appreciated the pledge, *p* = 0.253, and on the extent to which participants felt responsible for preventing sexual assault, *p* = 0.139.

As before, there were few main or interactive effects of gender and year of study. Two exceptions were that women were more likely to report that everyone should take the pledge, regardless of condition, and freshmen were more likely to report their friends’ unanimous appreciation of the pledge compared to all other students, regardless of condition (see Table 2 and Supplementary Materials).

**Problem of Sexual Assault:** We found a significant effect of treatment on whether participants considered sexual assault to be a problem on campus, B = −0.47, SE = 0.20, 95 % CI [−0.87, −0.08], *p* = 0.020. Those who read the moralistic pledge considered sexual assault to be less of a problem than those who read the informational pledge. There was also a significant effect of gender, B = −0.87, SE = 0.20, 95% CI [−1.25, −0.48], *p* < 0.001, such that women were more likely than men to consider sexual assault a problem.

**Party experience:** Consistent with Study 1, we did not find a main effect of condition on enjoyment of the party, feelings of safety at the party, and whether they would bring their little sister to the party (all at *p* > 0.510). However, we did find a gender effect, (B = 0.15, SE = 0.05, 95% CI [0.05, 0.26], *p* = 0.005), such that men reported feeling safer at the party than women did.

Taken together, the results of Study 2 suggest that exposure to the moralistic pledge decreased thinking about consent in moral terms, compared to the informational pledge; these results stand in contrast to those observed in Study 1.

## 9. Local Social Expectations: Contrasting Studies 1 and 2

**Figure 1:**
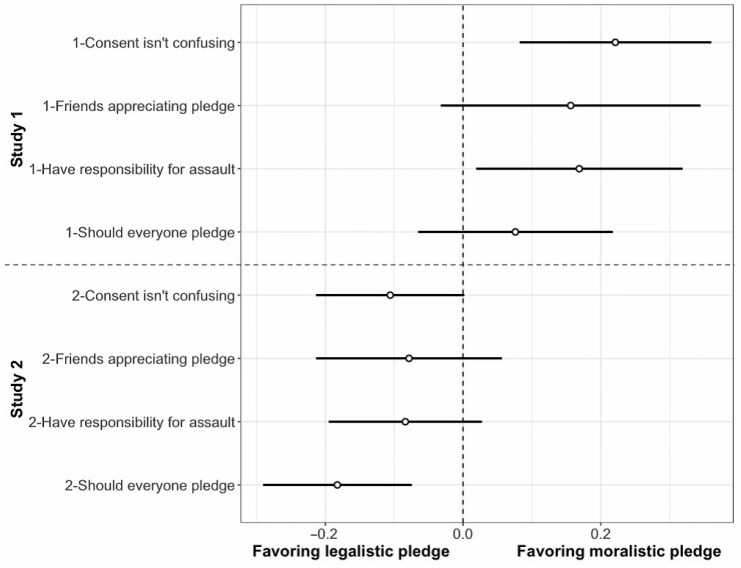
Responses to the moralistic and informational consent pledge by students at Club 1 and Club 2. More extreme values (i.e., greater deviation from zero) indicate more thinking about consent in moral terms.

Given evidence that the moralistic pledge significantly affected moral thinking in different directions at each club, we combined the data from Studies 1 and 2 to contrast the effects directly in an exploratory analysis (see Table 3). We found significant interaction effects between the treatment and the study setting for every variable we used to measure thinking about consent in moral terms; see Figure 1.

Specifically, after receiving the moralistic pledge, participants in Study 1 showed significantly stronger agreement with our moralized thinking items than participants in Study 2 regarding whether everyone should pledge, B = −0.26, SE = 0.09, 95% CI [−0.44, −0.08], *p* = 0.004; whether all of their friends appreciated the pledge, B = −0.23, SE = 0.12, 95% CI [−0.46, −0.01], *p* = 0.045; and whether they felt completely responsible for preventing sexual assault at their university, B = −0.25, SE = 0.10, 95% CI [−0.44, −0.07], *p* = 0.008. We found the same interaction pattern for the survey item testing whether consent was confusing, our reverse-coded item, interaction B = 0.33, SE = 0.09, 95% CI [0.15, 0.50], *p* < 0.001, such that participants at Club 1 reported less confusion about consent after reading the moralistic pledge and participants at Club 2 reported less confusion about consent when they received the informational pledge. We did not detect any differential interaction effects of the treatment on party experience dependent on context (all interaction effects at *p* > 0.42). The same experimental manipulation, presenting either a moralistic or informational message about consent, led to contrastive effects on moralized thinking at two social clubs at the same university. At Club 1, where an institutional consent practice already existed, exposure to the moralistic message led to relatively more moralized thinking about consent. At Club 2, without an existing consent practice, exposure to the moralistic message led to relatively less moralized thinking about consent.

## 10. Study 3

So far, we have provided evidence that exposure to moralistic, compared to informational, institutional language about consent can change how people think about consent, and that the institutional precedent for this language has differential consequences for moralization of the idea. We have not yet tested whether the effects of such language extend to behavioral change. Measuring behavior related to consent is difficult (DeGue et al., 2014; Porat et al., 2024), but it is the ultimate goal of any intervention about sexual consent. In a third study, we attempted to capture a variety of behaviors related to consent following a night in which consent pledges were administered at concurrent parties across all of the social clubs at the university. We find intriguing but weak results. We report them in the spirit of transparency and also as an example of one method for tying a campus-based intervention to behavioral data. Multiple meta-analyses in the field of sexual violence reduction underline the need for such methods (DeGue et al., 2014; Porat et al., 2024).

### 10.1. Method

#### Participants and Procedure

We targeted our investigation to an annual celebration of the close of the academic spring term, which students celebrate by hosting concurrent parties in all eleven of the campus’s social clubs. The night always falls on a date in mid-May, and parties last into the early morning hours. Leveraging our growing relationship with the clubs, we asked every club to implement some kind of messaging practice about consent on this night. The university keeps records of disciplinary events throughout the school year, which we were able to partially access as anonymized descriptions of behavioral infractions. We also fielded a team of researchers on campus that night to have brief conversations with students to casually assess people’s on-site reactions to the consent interventions and conducted 167 conversations in total.

All eleven clubs agreed to implement some kind of consent pledge at their door on the evening of 15 May. Our research team supplied the informational and moralistic pledges tested in the previous studies with individual clubs, but each club was free to choose or create its own message. Club 1 opted for the moralistic pledge; most other Clubs opted for the informational pledge and a few created their own that did not directly mention consent but referenced good behavior. The pledges were administered in a variety of ways. Some clubs requested that each student read the pledge aloud to enter, some allowed groups of students to read together as they entered, some gave their written pledge to students to read silently before entry, and one was hung on a wall in the club house. Given our working hypothesis that the effects of the consent pledge are sensitive to the local institutional context, we did not mandate that all clubs implement the consent pledge in the same way or with the same wording. The research team encouraged clubs to design the intervention in a way that suited their particular local institution. Not all clubs clearly communicated to us what they did or whether they did it for the entire night. Of note, while the leadership of each club was given guidance as to what the content of a consent pledge of their choosing could look like, we did not randomly assign students to read a moralistic pledge as a treatment. That is, students who entered each party all read the same pledge, but the content of each club’s pledge varied at the discretion of each club’s leadership.

### 10.2. Results

**Analytic approach:** To estimate the effect of this all-clubs consent pledge on a single night, we obtained anonymized administrative university records of student disciplinary actions and compared the number of incidents reported that night to those from the same celebration night held in the previous and subsequent years. This research was approved by the Princeton Institutional Review Board #12112 and the Institutional Review Panel for the use of Administrative Data in Research.

Specifically, we obtained data on all campus police reports and descriptions of undergraduate disciplinary infractions from 8 September 2007 to 30 August 2019. Given that the clubs’ pledges appealed to more than sexual consent and also included respect for other students at the party, we reasoned that the intervention could influence multiple types of disciplinary infractions. Over the span of the 13 years represented in our dataset, we thus included behaviors related to sexual harassment, assault, and/or indecent exposure (N = 153 total incidents over 13 years), harm towards others (N = 548), or engagement in other party-related infractions such as like urinating in public (N = 1765).

We classified infractions into these categories based on the administrative records’ written descriptions of each infraction. All coders first coded one year of infractions and then met to resolve disagreements. After this initial meeting, two of the four coders coded each of the rest of the years, such that each infraction had two raters, with varying pairs (so no two raters were consistently paired together). We then calculated inter-rater reliability (81.5%) and resolved all coding disagreements. Infractions related to other (i.e., not party-related) disciplinary behavior (e.g., shoplifting; unauthorized pets) were excluded (N = 904), which left us with 2049 total student infractions (1650 male) across 4375 days.

The night of campus-wide post-finals parties occurs 22 days before the start of summer term each year, so we examined whether significantly fewer infractions occurred during this 22-day time period, starting the night of the celebration and ending 22 days later. We contrasted the 15 May 2018–5 June 2018 period with comparable 22-day periods from other years included in the obtained dataset (i.e., 2007–2019). A significant treatment effect emerged, such that we observed fewer disciplinary infractions in the period following the campus pledge night compared to the same window of time following the campus parties in all other years available, B = −0.55, SE = 0.14, 95% CI [−0.84, −0.27], *p* < 0.001, controlling for infractions during the celebration night each year prior, the yearly period of 22 days following the campus-wide party night, the pledge night itself, recess periods (Spring, Summer, Fall, Thanksgiving, and Winter break), and fixed effects for academic year to account for variations in the number of infractions between school years.

Nonetheless, we contend that these results are suggestive but inconclusive. For one, our model is not robust to alternative specifications. A test of the difference between infractions in the post-pledge night period vs. the same period in all other years was not significant without robust standard errors, *p* = 0.130. Second, although we observe a significant reduction in infractions in the full time window of the pledge and the subsequent 22 days, we find a significant increase in infractions on the night of the pledge itself compared to other years B = 4.37, SE = 0.65, 95% CI [3.10, 5.64], *p* < 0.001. This increase in infractions did not include more reporting of sexual assault, but rather, a greater number of reported alcohol-related infractions.

**Qualitative responses:** We also examined student comments on the all-club pledge night from our brief interviews with students on the street. These interviews were not randomly solicited, so responses reflect reactions from students who were willing to talk to researchers on the street outside of the clubs. Students reported an overall positive evaluation of the consent intervention, with some skepticism of its utility, and we see evidence that different groups of students may be talking about it in different ways (see Table 4 for representative quotations).

## 11. General Discussion

How do institutional messages shape processes of moralization? These experiments make progress toward this broad theoretical question by testing the causal effects of a real-world intervention that randomized whether people received a moralistic or informational message about consent in local institutional settings that vary in the extent to which members have signaled their commitment to the issue and imbued it with moral significance. We conducted the same experiment in two different student clubs, allowing us to explore how institutional settings that differ in their signaling of consent as a moral value may interact with moral messaging at different strengths to affect individual-level moralization. Our results suggest that moralistic messages do not yield uniform responses. Instead, we find that in a context where there is an institutionalized practice highlighting the importance of consent, a moralistic message (vs. an informational message) engages students’ thinking about consent in moral terms, possibly triggering what we now would call moral amplification. In contrast, in a party setting organized by a student group with no institutionalized practice regarding consent, students reported greater moralized thinking about consent in response to an informational message about the practice, which did not portray it as a moral issue, suggesting that here, the issue of consent was not already widely moralized. We observe a significant interaction between the two social clubs and the moralistic vs. informational messages in subsequent exploratory analyses.

One interpretation of these findings is that the existing institutionalized practice of the consent pledge at Club 1 reflects greater widespread moral recognition among party attendees at Club 1, whereas the lack of institutional practice at Club 2 might reflect more variation in the moral recognition of sexual consent among Club 2’s party-goers. This raises the intriguing possibility that explicitly moralistic messages are less effective in places where moralized attitudes are less widespread or less uniform—or that moralistic messages are not as effective for moral recognition as they are for moral amplification. Moreover, these contrastive effects between two groups within one university campus are consistent with the idea that social mechanisms, like local institutional and peer signaling of values, play an important role in changing individual-level moralization of an idea. We attempted to connect this messaging to real-world behavior (incidents on campus that were captured by the university or by campus police) and we found evidence of a decrease in party-related disciplinary incidents on campus in the 22 days following the all-clubs pledge night compared to the same time period in years before and after. This evidence is suggestive but inconclusive given that (a) the results are not significant without robust standard errors and (b) we observed a significant increase in (alcohol-related) infractions on the night of the pledge itself.

### 11.1. Theoretical Implications

At the time that we designed and conducted these experiments (from Fall 2016–Spring 2018), we did not have the helpful theoretical categories of moral recognition and moral amplification (Rhee et al., 2019) to ground and inform our empirical work. The present data in both studies and club contexts were heavily left-skewed (in that study participants’ responses were on higher ends of the scale), indicating that the majority of participants in both contexts tended to show strong agreement with consent as positively moralized (i.e., an imperative, normative, responsibility). However, our contrasting effects in the two social clubs suggest that there may be important social preconditions to moral amplification. Specifically, it is possible that in the institution that had already institutionalized a practice around consent established norms that facilitated individuals increasingly thinking about consent in moral terms—or moral amplification. It is also possible that at the other club, at least some of the individuals at the party did not recognize sexual consent as a moral issue (though they may still see it as an important or normative one).

### 11.2. Policy Relevance and Future Directions

Our findings have important implications for crafting messages about sexual consent and other issues whose moral status may be unknown or in flux. Our context-dependent effects suggest that messages should be tailored to match the extent to which a local institutional group has moralized an issue, rather than dispensed uniformly from a larger institution—even for groups that belong to the same larger cultural context such as the same university. Different social groups establish their own practices that promote certain ideas or issues as more normative, as imperatives to action, and as clear matters. In short, different group practices promote certain ideas as more moralized. Importantly, groups may differ not only in the degree to which their members may moralize an issue but also in the extent to which their members recognize an issue as moral. Future research should more explicitly test messages aimed at increasing or decreasing moral recognition and moral amplification as separate processes. Finally, much prior work on moralization has focused on meat eating, cigarette smoking, and abortion. As far as we know, this work is the first to examine moralization of sexual consent. Moreover, we examine positive moralization, that is, regarding sexual consent as morally good, rather than negative moralization or moral opposition, regarding sexual misconduct as morally bad. As far as we know, the present study is the first of its kind to examine moralization processes of an issue that has a positive moral valence. Future research would benefit from careful attention to whether institutional signals can change positive and negative moralization in similar ways.

Pragmatically, these findings are significant for those seeking to boost (or reduce) the perceived moral status of a societal issue. Universities often use messages that either directly or indirectly moralize consent and sexual assault in the context of student and staff trainings, operating under the assumption that the message will be perceived similarly across various university subgroups. The present study offers two ways to rethink this strategy. The first is to consider different messages for different subgroups, since our evidence suggests that even very similar-seeming groups (students who belong to social clubs and who attend late-night parties) may respond differently to moralistic messages about consent. The second is to consider engaging these subgroups directly by asking for input and encouraging them to lead messaging efforts. We think these findings are particularly illuminating because a priori it might be tempting for people or institutions who want to promote the moralization of consent to use moralistic messages in a top-down manner, particularly in places where they may speculate it is “most needed” or among groups where there is relatively less recognition of sexual consent as a moral issue. Our empirical results suggest that this will not be uniformly effective and that other types of messages, like informational ones, may be a more effective starting point in some contexts.

Future work aimed at replicating and scaling up these findings should replicate the process, rather than the content, of the intervention tested here. In other words, we do not suggest that future researchers use the precise content of the pledges in this study but rather attempt to replicate the process of how this community practice came about. Students in this study’s context selected the content and mode of intervention, and their efforts were seen as an initiative of their local institution—the social club. Other important aspects of this process may include ensuring group buy-in, making the practice public, and having key members of the group be the purveyors of the message. This is especially important given that this research took place in a specific context—an elite American university that has neither a nationally representative student body nor an international community large enough to warrant broad claims about generalizability.

We also investigated whether one night of an all-clubs consent pledge could affect student behavior. We used anonymized administrative university data of all campus police reports and descriptions of undergraduate disciplinary infractions from 8 September 2007 to 30 August 2019, and collected informal interview responses to capture the effect of the one-night campus-wide consent practice. We do not find robust effects, but these methods for measuring behavior are worth the attempt. Measuring observed behaviors in the field of sexual violence is challenging, and innovations are needed even if they are imperfect (DeGue et al., 2014; Porat et al., 2024).

### 11.3. Limitations

In general, it is difficult to disentangle an institution or a context from the people who comprise it. Where did these effects come from? On the one hand, it is possible that different students attended the two parties and the traits of these different students may account for the different effects observed at each club. On the other hand, the two different environments may promote different reactions to the consent pledges, even if the students are the same. With our present experimental design, we cannot adjudicate between these explanations—this is a limitation of the studies. Moreover, Club 1 mandated that students take the pledge, while Club 2 did not. Requiring the pledge may itself signal that consent was already more likely to be thought about in positive moral terms at Club 1 than at Club 2. At Club 2, 19 out of 538 (3.5% of) party attendees opted out of the pledge by walking past the research team or verbally opting out. Though this is a relatively low drop-out rate, it may indicate that there was more variation in the moral status of consent among party attendees at Club 2 than at Club 2.

An additional limitation of the present work is that we do not know the full extent of which cognitive processes were initiated by students who interacted with the pledge. Our moralistic pledge intentionally invoked multiple constructs that we thought would be important for the pledge to resonate with students, including social identity, moral values, and the invocation of norms. We did this to maximize the impact of the pledge on the minds and behaviors of students in this unique context. We call it a moralistic pledge here because the pledge most strongly invokes moral values, which are often tied to social identity (Ellemers et al., 2017) and to social norms (Lindström et al., 2018) using moralized language (Gantman & Van Bavel, 2015). Given that we emphasize here that the process, rather than the content, of constructing the intervention is most important, we think that the precise mechanism is less important than the overall impact.

Furthermore, we simply do not know how long the effects of the different consent pledges last. Do the pledges affect behavior in the immediate context only, with short-term effects? This would mean that individuals influenced to think about consent in moral terms do so depending on the place they are in. It is possible that the pledges could have longer-lasting individual difference effects. This could result from people who belong to the community internalizing the value of consent after they take the pledge multiple times due to regular party attendance. Or a longer-lasting effect could result from people pledging for the first time and having a notable and durable experience as a result. Because our data cannot tell us whether the effect is long-lasting and why, the safer assumption for now is that the pledge results in a short-term, more contextually bound effect. Future research should test the importance of the repetition of these community practices. We do not know how important it is that Club 1 routinely used their consent pledge before collaborating on an experiment of it while the second club was new to the pledge.

Finally, another limitation of the present research is that we did not include a “no pledge” control group. Club 1 in Experiment 1 was not comfortable with removing the pledge for some party attendees, and we respected their decision. The benefit of a no-pledge control would be to assess whether, for example in Study 2, students merely preferred the informational pledge or were reacting negatively to the moralistic one. Additionally, the presence of such a condition might better elucidate the extent to which people tended to moralize consent without receiving any information about it—which could be used to more precisely estimate whether and to what extent participants underwent processes of moral recognition (i.e., updating whether they think sexual consent is a moral issue or not) or moral amplification (i.e., to what extent they regard sexual consent as a moral issue) after interacting with the consent pledges. Future research would benefit from disentangling preferences for non-moralistic messages vs. reactance to moralistic ones both in communities where moral recognition of the issue is widespread and in ones where it is not.

### 11.4. Conclusions

Our research highlights the importance of considering individual-level moralization as a contextually based process, sensitive to the messages coming from local institutions. We find evidence that people moralize sexual consent to a different extent after reading a moralistic message about consent at the door of a party, depending on where the party is held. These two levels of moralization, at the individual level and the local institutional level, may interact to facilitate individual and group processes of moralization and behavior change.

## Figures and Tables

**Table 1:** Treatment effect of moralistic pledge at Eating Club 1.

	Universality *Should Everyone Pledge?*		Perceived Norms *What Percent of Your Friends Appreciate the Pledge?*		Clarity of Consent *Is Consent Confusing?*		Responsibility *How Responsible Are You for Preventing Sexual Assault?*	
	(1)	(2)	(3)	(4)	(5)	(6)	(7)	(8)
Pledge Type	0.08 (0.07) [−0.07, 0.22] {0.291}	0.08 (0.07) [−0.06, 0.23] {0.270}	0.16 (0.09) [−0.03, 0.34] {0.102}	0.12 (0.10) [−0.08, 0.32] {0.251}	−0.22 ** (0.07) [−0.36, −0.08] {0.002}	−0.24 ** (0.07) [−0.38, −0.10] {0.0012}	0.17 * (0.08) [0.02, 0.32] {0.028}	0.21 ** (0.08) [0.06, 0.36] {0.008}
Gender		−0.06 (0.08) [−0.21, 0.09] {0.419}		−0.10 (0.10) [−0.30, 0.10] {0.339}		0.004 (0.07) [−0.14, 0.15] {0.958}		−0.04 (0.08) [−0.20, 0.12] {0.647}
Freshman Status		−0.17 * (0.07) [−0.31, −0.02] {0.025}		0.05 (0.11) [−0.16, 0.27] {0.339}		−0.08 (0.08) [−0.23, 0.08] {0.328}		−0.15 (0.08) [−0.31, 0.01] {0.058}
Constant	0.62 *** (0.05) [0.52, 0.72]	0.78 *** (0.08) [0.61, 0.94]	0.33 *** (0.07) [0.19, 0.46]	0.36 ** (0.12) [0.13, 0.59]	−0.54 *** (0.05) [−0.65, −0.43]	−0.51 *** (0.09) [−0.68, −0.33]	0.46 *** (0.06) [0.35, 0.57]	0.58 *** (0.09) [0.39, 0.76]
Adj. *R*^2^	0.01	0.03	0.03	0.03	0.05	0.08	0.03	0.06
*N*	176	160	107	101	175	159	169	154

*Note:* Coefficients are regression coefficients. Standard errors are provided in parentheses, 95% confidence intervals are provided in brackets, and *p* values are provided in curly brackets. * *p* < 0.05, ** *p* < 0.01, *** *p* < 0.001.

**Table 2:** Treatment effect of moralistic pledge at Eating Club 2.

	Universality *Should Everyone Pledge?*		Perceived Norms *What Percent of Your Friends Appreciate the Pledge?*		Clarity of Consent *Is Consent Confusing?*		Responsibility *How Responsible Are You for Preventing Sexual Assault?*	
	(1)	(2)	(3)	(4)	(5)	(6)	(7)	(8)
Pledge Type	−0.18 ** (0.05) [−0.29, −0.07] {0.001}	−0.19 *** (0.06) [−0.31, −0.08] {0.0006}	−0.08 (0.07) [−0.21, 0.06] {0.253}	−0.10 (0.07) [−0.24, 0.04] {0.157}	0.11 (0.05) [−0.002, 0.21] {0.054}	0.10 (0.06) [−0.02, 0.21] {0.093}	−0.08 (0.06) [−0.19, 0.03] {0.139}	−0.10 (0.06) [−0.21, 0.02] {0.099}
Gender		−0.18 ** (0.06) [−0.29, −0.07] {0.002}		0.04 (0.07) [−0.10, 0.18] {0.600}		0.01 (0.06) [−0.10, 0.12] {0.862}		−0.01 (0.06) [−0.13, 0.10] {0.808}
Freshman Status		−0.01 (0.06) [−0.13, 0.10] {0.843}		−0.16 * (0.08) [−0.32, −0.01] {0.037}		0.05 (0.06) [−0.07, 0.17] {0.413}		−0.11 (0.06) [−0.23, 0.01] {0.064}
Constant	0.68 *** (0.04) [0.61, 0.75]	0.78 *** (0.06) [0.67, 0.90]	0.46 *** (0.05) [0.36, 0.55]	0.56 *** (0.08) [0.41, 0.72]	−0.68 *** (0.04) [−0.75, −0.60]	−0.70 *** (0.06) [−0.82, −0.58]	0.59 *** (0.04) [0.52, 0.67]	0.68 *** (0.06) [0.56, 0.80]
Adj. *R*^2^	0.03	0.08	0.01	0.04	0.01	0.01	0.01	0.02
*N*	312	292	208	194	312	293	310	290

*Note:* Coefficients are regression coefficients. Standard errors are provided in parentheses, 95% confidence intervals are provided in brackets, and *p* values are provided in curly brackets. * *p* < 0.05, ** *p* < 0.01, *** *p* < 0.001.

**Table 3:** Context (Eating Club 1 vs. 2) X treatment (legalistic vs. moralistic pledge) effects.

	Universality *Should Everyone Pledge?*		Perceived Norms *What Percent of Your Friends Appreciate the Pledge?*		Clarity of Consent *Is Consent Confusing?*		Responsibility *How Responsible Are You for Preventing Sexual Assault?*	
	(1)	(2)	(3)	(4)	(5)	(6)	(7)	(8)
Context	0.06 (0.06) [−0.07, 0.18] {0.362}	0.04 (0.07) [−0.09, 0.17] {0.571}	0.13 (0.08) [−0.03, 0.29] {0.118}	0.13 (0.09) [−0.04, 0.31] {0.130}	−0.14 * (0.07) [−0.27, −0.01] {0.037}	−0.11 (0.07) [−0.25, 0.02] {0.099}	−13 (0.07) [−0.004, 0.26] {0.056}	0.14 * (0.07) [0.002, 0.28] {0.047}
Pledge Type	0.08 (0.07) [−0.06, 0.22] {0.289}	0.07 (0.07) [−0.07, 0.22] {0.317}	0.16 (0.09) [−0.03, 0.34] {0.099}	0.14 (0.10) [−0.06, 0.34] {0.163}	−0.22 ** (0.07) [−0.36, −0.08] {0.002}	−0.25 *** (0.07) [−0.39, −0.11] {0.001}	0.17 * (0.08) [0.02, 0.32] {0.027}	0.21 ** (0.08) [0.05, 0.36] {0.008}
Context X Pledge Type	−0.26 ** (0.09) [−0.44, −0.08] {0.004}	−0.27 ** (0.09) [−0.45, −0.09] {0.004}	−0.23 * (0.12) [−0.46, −0.01] {0.045}	−0.24 (0.12) [−0.48, −0.002] {0.052}	0.33 *** (0.09) [0.15, 0.50] {0.0003}	0.35 *** (0.09) [0.17, 0.53] {0.0002}	−0.25 ** (0.09) [−0.44, −0.07] {0.008}	−0.30 ** (0.10) [−0.49, −0.11] {0.002}
Gender		−0.14 ** (0.05) [−0.23, −0.05] {0.003}		−0.01 (0.06) [−0.12, 0.10] {0.859}		0.01 (0.05) [−0.08, 0.10] {0.866}		−0.02 (0.05) [−0.12, 0.07] {0.641}
Freshman Status		−0.07 (0.05) [−0.16, 0.02] {0.149}		−0.09 (0.06) [−0.22, 0.03] {0.157}		0.005 (0.05) [−0.09, 0.10] {0.918}		−0.13 ** (0.05) [−0.22, −0.03] {0.009}
Constant	0.62 *** (0.05) [0.52, 0.72]	0.76 *** (0.07) [0.63, 0.90]	0.33 *** (0.07) [0.19, 0.46]	0.40 *** (0.09) [0.23, 0.57]	−0.54 *** (0.05) [−0.65, −0.43]	−0.56 *** (0.7) [−0.70, −0.42]	0.46 *** (0.06) [0.35, 0.57]	0.55 *** (0.07) [0.41, 0.69]
Adj. *R*^2^	0.03	0.06	0.01	0.02	0.03	0.03	0.01	0.04
*N*	488	452	315	295	487	452	479	444

*Note:* Coefficients are regression coefficients. Standard errors are provided in parentheses, 95% confidence intervals are provided in brackets, and *p* values are provided in curly brackets. * *p* < 0.05, ** *p* < 0.01, *** *p* < 0.001.

**Table 4:** Student responses to one-night campus-wide consent practice.

**What do you remember of [the pledge]? What do you think about it (or about consent more generally)?**
“It was really annoying … long. I don’t know what I read, it was so long. It’s a good “thing” … but I like wasn’t even thinking about it, like no one cares, it doesn’t really matter.”
“I think it’s a good idea, they have been increasing it … wonder if it actually changes anything. But it can’t hurt, if it helps change 1 person that is worth it.”
“Mindless for most people.”
“Better than nothing.”
**How do you think other people are reacting to it? Will people be talking about it tomorrow?**
“Not any real reaction. People are talking about it, but not in a negative way. But that is good, the pledge brings discourse about these issues that you wouldn’t normally talk about.”
“No talk. It is just a formality.”
“Every conversation has been positive.”
“Split—a subset who fill find it excessive but most will be supportive.”
“People were talking about it tonight so probably we’ll talk about it tomorrow”

## Data Availability

All data, pre-registrations, and code are available on the project’s OSF page (https://osf.io/w7btc/) with one exception. We analyzed administrative-level conduct complaints given to us by Princeton University. As part of our agreement to use the data, we are not permitted to make it public (6 July 2019).

## Notes

1 This includes pre-registration of a pilot study at Club 1, which we used to test and adjust our design and measures. Because the measures differed from those used in the two main studies, we do not discuss the pilot here.

2 The normativity item used a 1–100 scale; therefore, it was analyzed separately. Consent was most highly moralized (M = 84, SD = 18.95), followed by infidelity in marriage (M = 77.63, SD = 22.05) and online dating (M = 59.8, SD = 24.08). The differences between averages for this item were all statistically significant (all at *p* < 0.001).

## Supplementary Materials

The following supporting information can be downloaded at: https://www.mdpi.com/article/10.3390/bs15081025/s1, Table S1: Correlations between face-valid measures of moral judgments and consent survey items; Table S2: Correlations between face-valid measures of moral judgments and infidelity survey items; Table S3: Correlations between face-valid measures of moral judgments and online dating survey items; Table S4: Cronbach’s alpha for moralization of different topics; Table S5: Means: 0–4 scale item indexes; Table S6: Means: 1–100 scale item; Table S7: Comparisons: 0–4 scale item indexes; Table S8: Comparisons: 0–100 scale items; Table S9: Responsibility: How responsible are you for preventing sexual assault?; Table S10: Universality: Should everyone pledge?; Table S11: Perceived norms: What % of your friends appreciate the pledge?; Table S12: Clarity of consent: Is consent confusing?; Table S13: Did you enjoy the party?; Table S14: Do you feel safe at this party?; Table S15: If you could, would you bring your little sister to this party?; Table S16: Responsibility: How responsible are you for preventing sexual assault?; Table S17: Universality: Should everyone pledge?; Table S18: Perceived norms: What % of your friends appreciate the pledge?; Table S19: Clarity of consent: Is consent confusing?; Table S20: Did you enjoy the party?; Table S21: Do you feel safe at this party?; Table S22: If you could, would you bring your little sister to this party?; Table S23: Is sexual assault a problem at [University]?; Table S24: Responsibility: How responsible are you for preventing sexual assault?; Table S25: Universality: Should everyone pledge?; Table S26: Perceived norms: What % of your friends appreciate the pledge?; Table S27: Clarity of consent: Is consent confusing?; Table S28: Did you enjoy the party?; Table S29: Do you feel safe at this party?; Table S30: If you could, would you bring your little sister to this party?
