# Supplementary material for: Moralizing Consent: Three Field Studies Testing a Student-Led Intervention at University Parties
Source: Behav Sci (Basel). 2025 Jul 29;15(8):1025. doi: 10.3390/bs15081025 (PMC12382995; doi:10.3390/bs15081025)
Supplement: Supplementary file 1 [file behavsci-15-01025-s001.zip › behavsci-3662773-supplementary.pdf]

Supplemental materials for: Moralizing Consent: Three Field Studies Testing A Student-Led Intervention at University Parties

Ana P. Gantman[1,2], Ajua Duker[3], Jordan Starck[4], Alex Sanchez[5], & Elizabeth Levy Paluck[5,6]

[1]Department of Psychology, Brooklyn College, City University of New York
[2]Department of Psychology, CUNY Graduate Center
[3]Department of Psychology, New York University
[4]Department of Psychology, Stanford University
[5]Department of Psychology, Princeton University
[6]School of Public and International Affairs, Princeton University

July 21, 2025

1 Construct Validation

Table S1: Correlations between face-valid measures of moral judgments and consent survey items

	cons_confuseR	cons_importance	cons_importanceD	cons_responsible	cons_every1	cons_fundamental	cons_core
cons_confuseR							
cons_importance	0.52***						
cons_importanceD	0.52***	0.61***					
cons_responsible	0.17*	0.21**	0.12				
cons_every1	0.44***	0.60***	0.40***	0.31***			
cons_fundamental	0.40***	0.45***	0.24**	0.22**	0.54***		
cons_core	0.41***	0.49***	0.25**	0.33***	0.44***	0.61***	

Table S2: Correlations between face-valid measures of moral judgments and infidelity survey items

	mono_confuseR	mono_importance	mono_importanceD	mono_responsible	mono_every1	mono_fundamental	mono_core
mono_confuseR							
mono_importance	0.32***						
mono_importanceD	0.28***	0.46***					
mono_responsible	0.15	0.29***	0.19*				
mono_every1	0.29***	0.33***	0.24**	0.35***			
mono_fundamental	0.30***	0.39***	0.11	0.39***	0.54***		
mono_core	0.20*	0.48***	0.23**	0.47***	0.47***	0.61***	

Table S3: Correlations between face-valid measures of moral judgments and online dating survey items

	date_confuseR	date_importance	date_importanceD	date_responsible	date_every1	date_fundamental	date_core
date_confuseR							
date_importance	-0.18*						
date_importanceD	0.12	0.30***					
date_responsible	-0.31***	0.20*	-0.15				
date_every1	-0.36***	0.39***	0.01	0.41***			
date_fundamental	-0.53***	0.18*	-0.10	0.45***	0.48***		
date_core	-0.49***	0.23**	-0.12	0.53***	0.42***	0.60***	

Table S4: Cronbach's alpha for moralization of different topics

Topic	Alpha
Consent	0.74
Fidelity in marriage	0.72
Online dating	0.78

Table S5: Means: 0-4 scale item indexes

Topic	Mean	SD	Min	Max
Consent	3.16	0.73	0	4
Fidelity in marriage	3.09	0.77	0	4
Online dating	1.98	0.72	0	4

Table S6: Means: 1-100 scale item

Topic	Mean	SD	Min	Max
Consent	3.16	0.73	0	4
Fidelity in marriage	3.09	0.77	0	4
Online dating	1.98	0.72	0	4

Table S7: Comparisons: 0-4 scale item indexes

Test	M	t	p	CI
Consent vs. fidelity	0.08	1.37	0.172	[-0.04, 0.20]
Consent vs. online dating	1.18	12.99	1.008946e-26	[1.00, 1.36]
fidelity vs. online dating	1.11	12.03	4.547053e-24	[0.93, 1.29]

Table S8: Comparisons: 0-100 scale items

Test	M	t	p	CI
Consent vs. fidelity	6.59	3.53	5.521957e-04	[2.90, 10.28]
Consent vs. online dating	24.13	9.37	7.211941e-17	[19.04, 29.21]
fidelity vs. online dating	17.91	6.93	9.90828e-11	[12.80, 23.01]

2 Study 1

Note: Coefficients are regression coefficients. Standard errors are provided in parentheses and 95% confidence intervals are provided in brackets. *$p < .05$, **$p < .01$, ***$p < .001$.

Table S9: Responsibility: How responsible are you for preventing sexual assault?

	(1)	(2)	(3)
Pledge Type	0.17* (0.08)	0.21** (0.08)	0.26 (0.16)
	[0.02, 0.32]	[0.06, 0.36]	[−0.06, 0.59]
Gender		−0.04 (0.08)	−0.08 (0.12)
		[−0.20, 0.12]	[−0.32, 0.16]
Freshman Status		−0.15 (0.08)	−0.08 (0.12)
		[−0.31, 0.01]	[−0.32, 0.16]
Pledge Type X Gender			0.09 (0.16)
			[−0.23, 0.41]
Pledge Type X Freshman Status			−0.17 (0.16)
			[−0.48, 0.15]
Constant	0.46*** (0.06)	0.58*** (0.09)	0.55*** (0.13)
	[0.35, 0.57]	[0.39, 0.76]	[0.30, 0.80]
Num.Obs.	169	154	154
R2	0.029	0.059	0.068
R2 Adj.	0.023	0.041	0.037
AIC	244.7	222.0	224.5
BIC	254.1	237.2	245.8
RMSE	0.49	0.48	0.48

Table S10: Universality: Should everyone pledge?

	(1)	(2)	(3)
Pledge Type	0.08 (0.07)	0.08 (0.07)	0.22 (0.14)
	[−0.07, 0.22]	[−0.06, 0.23]	[−0.07, 0.50]
Gender		−0.06 (0.08)	0.02 (0.11)
		[−0.21, 0.09]	[−0.21, 0.24]
Freshman Status		−0.17* (0.07)	−0.13 (0.11)
		[−0.31, −0.02]	[−0.35, 0.09]
Pledge Type X Gender			−0.15 (0.15)
			[−0.45, 0.15]
Pledge Type X Freshman Status			−0.06 (0.15)
			[−0.35, 0.23]
Constant	0.62*** (0.05)	0.78*** (0.08)	0.71*** (0.12)
	[0.52, 0.72]	[0.61, 0.94]	[0.47, 0.94]
Num.Obs.	176	160	160
R2	0.006	0.035	0.041
R2 Adj.	0.001	0.016	0.010
AIC	241.6	217.3	220.2
BIC	251.1	232.6	241.7
RMSE	0.47	0.46	0.46

Table S11: Perceived norms: What % of your friends appreciate the pledge?

	(1)	(2)	(3)
Pledge Type	0.16 (0.09)	0.12 (0.10)	0.29 (0.23)
	[−0.03, 0.34]	[−0.08, 0.32]	[−0.16, 0.74]
Gender		−0.10 (0.10)	0.00 (0.15)
		[−0.30, 0.10]	[−0.29, 0.29]
Freshman Status		0.05 (0.11)	0.10 (0.15)
		[−0.16, 0.27]	[−0.19, 0.39]
Pledge Type X Gender			−0.18 (0.20)
			[−0.58, 0.23]
Pledge Type X Freshman Status			−0.10 (0.22)
			[−0.53, 0.34]
Constant	0.33*** (0.07)	0.36** (0.12)	0.28 (0.15)
	[0.19, 0.46]	[0.13, 0.59]	[−0.02, 0.58]
Num.Obs.	107	101	101
R2	0.025	0.028	0.037
R2 Adj.	0.016	−0.002	−0.014
AIC	155.2	150.1	153.2
BIC	163.2	163.2	171.5
RMSE	0.49	0.48	0.48

Table S12: Clarity of consent: Is consent confusing?

	(1)	(2)	(3)
Pledge Type	−0.22** (0.07)	−0.24** (0.07)	−0.44** (0.15)
	[−0.36, −0.08]	[−0.38, −0.10]	[−0.74, −0.13]
Gender		0.00 (0.07)	−0.08 (0.12)
		[−0.14, 0.15]	[−0.31, 0.15]
Freshman Status		−0.08 (0.08)	−0.15 (0.12)
		[−0.23, 0.08]	[−0.38, 0.08]
Pledge Type X Gender			0.16 (0.15)
			[−0.13, 0.46]
Pledge Type X Freshman Status			0.14 (0.15)
			[−0.16, 0.45]
Constant	−0.54*** (0.05)	−0.51*** (0.09)	−0.41*** (0.12)
	[−0.65, −0.43]	[−0.68, −0.33]	[−0.65, −0.17]
Num.Obs.	175	159	159
R2	0.054	0.076	0.087
R2 Adj.	0.048	0.058	0.057
AIC	233.5	208.0	210.1
BIC	243.0	223.4	231.6
RMSE	0.46	0.45	0.45

Table S13: Did you enjoy the party?

	(1)	(2)	(3)
Pledge Type	−0.02 (0.07)	0.02 (0.08)	0.16 (0.16)
	[−0.16, 0.13]	[−0.13, 0.17]	[−0.16, 0.49]
Gender		−0.01 (0.08)	0.10 (0.10)
		[−0.16, 0.15]	[−0.10, 0.31]
Freshman Status		−0.03 (0.08)	−0.02 (0.11)
		[−0.19, 0.12]	[−0.23, 0.19]
Pledge Type X Gender			−0.22 (0.15)
			[−0.53, 0.08]
Pledge Type X Freshman Status			−0.01 (0.16)
			[−0.31, 0.30]
Constant	0.32*** (0.05)	0.32*** (0.09)	0.25* (0.10)
	[0.22, 0.42]	[0.15, 0.50]	[0.05, 0.45]
Num.Obs.	169	156	156
R2	0.000	0.002	0.015
R2 Adj.	−0.006	−0.018	−0.018
AIC	226.0	211.2	213.1
BIC	235.4	226.5	234.5
RMSE	0.46	0.46	0.46

Table S14: Do you feel safe at this party?

	(1)	(2)	(3)
Pledge Type	0.02 (0.07)	0.02 (0.07)	0.19 (0.15)
	[−0.12, 0.15]	[−0.12, 0.16]	[−0.12, 0.49]
Gender		0.08 (0.08)	0.12 (0.11)
		[−0.06, 0.23]	[−0.10, 0.34]
Freshman Status		−0.07 (0.07)	0.02 (0.11)
		[−0.21, 0.07]	[−0.19, 0.24]
Pledge Type X Gender			−0.06 (0.15)
			[−0.36, 0.24]
Pledge Type X Freshman Status			−0.19 (0.14)
			[−0.47, 0.09]
Constant	0.73*** (0.05)	0.73*** (0.09)	0.65*** (0.12)
	[0.64, 0.83]	[0.56, 0.90]	[0.41, 0.88]
Num.Obs.	167	154	154
R2	0.000	0.017	0.028
R2 Adj.	−0.006	−0.003	−0.005
AIC	203.6	188.0	190.2
BIC	212.9	203.2	211.4
RMSE	0.44	0.43	0.43

Table S15: If you could, would you bring your little sister to this party?

	(1)	(2)	(3)
Pledge Type	0.07 (0.07)	0.07 (0.08)	0.27 (0.17)
	[−0.07, 0.22]	[−0.09, 0.22]	[−0.07, 0.61]
Gender		−0.06 (0.08)	0.01 (0.11)
		[−0.22, 0.10]	[−0.22, 0.23]
Freshman Status		0.06 (0.08)	0.16 (0.11)
		[−0.10, 0.22]	[−0.05, 0.38]
Pledge Type X Gender			−0.12 (0.16)
			[−0.44, 0.20]
Pledge Type X Freshman Status			−0.20 (0.16)
			[−0.52, 0.12]
Constant	0.30*** (0.05)	0.32*** (0.09)	0.22* (0.11)
	[0.20, 0.41]	[0.15, 0.49]	[0.00, 0.44]
Num.Obs.	167	154	154
R2	0.006	0.014	0.026
R2 Adj.	0.000	−0.006	−0.007
AIC	229.7	218.3	220.4
BIC	239.1	233.5	241.6
RMSE	0.47	0.48	0.47

3 Study 2

Table S16: Responsibility: How responsible are you for preventing sexual assault?

	(1)	(2)	(3)
Pledge Type	−0.08 (0.06)	−0.10 (0.06)	−0.12 (0.12)
	[−0.19, 0.03]	[−0.21, 0.02]	[−0.36, 0.11]
Gender		−0.01 (0.06)	−0.02 (0.08)
		[−0.13, 0.10]	[−0.18, 0.14]
Freshman Status		−0.11 (0.06)	−0.13 (0.08)
		[−0.23, 0.01]	[−0.29, 0.04]
Pledge Type X Gender			0.02 (0.12)
			[−0.21, 0.25]
Pledge Type X Freshman Status			0.02 (0.12)
			[−0.22, 0.27]
Constant	0.59*** (0.04)	0.68*** (0.06)	0.69*** (0.08)
	[0.52, 0.67]	[0.56, 0.80]	[0.54, 0.84]
Num.Obs.	310	290	290
R2	0.007	0.023	0.023
R2 Adj.	0.004	0.012	0.006
AIC	450.5	421.6	425.5
BIC	461.7	439.9	451.2
RMSE	0.50	0.49	0.49

Table S17: Universality: Should everyone pledge?

	(1)	(2)	(3)
Pledge Type	−0.18** (0.05)	−0.19*** (0.06)	−0.13 (0.12)
	[−0.29, −0.07]	[−0.31, −0.08]	[−0.36, 0.09]
Gender		−0.18** (0.06)	−0.17* (0.08)
		[−0.29, −0.07]	[−0.33, −0.02]
Freshman Status		−0.01 (0.06)	0.03 (0.08)
		[−0.13, 0.10]	[−0.13, 0.19]
Pledge Type X Gender			−0.01 (0.11)
			[−0.23, 0.21]
Pledge Type X Freshman Status			−0.09 (0.12)
			[−0.32, 0.15]
Constant	0.68*** (0.04)	0.78*** (0.06)	0.76*** (0.07)
	[0.61, 0.75]	[0.67, 0.90]	[0.61, 0.90]
Num.Obs.	312	292	292
R2	0.034	0.078	0.080
R2 Adj.	0.031	0.069	0.064
AIC	437.8	401.4	404.9
BIC	449.0	419.8	430.6
RMSE	0.48	0.47	0.47

Table S18: Perceived norms: What % of your friends appreciate the pledge?

	(1)	(2)	(3)
Pledge Type	−0.08 (0.07)	−0.10 (0.07)	−0.22 (0.15)
	[−0.21, 0.06]	[−0.24, 0.04]	[−0.51, 0.07]
Gender		0.04 (0.07)	0.03 (0.10)
		[−0.10, 0.18]	[−0.17, 0.23]
Freshman Status		−0.16* (0.08)	−0.24* (0.11)
		[−0.32, −0.01]	[−0.45, −0.03]
Pledge Type X Gender			0.02 (0.14)
			[−0.26, 0.30]
Pledge Type X Freshman Status			0.16 (0.15)
			[−0.15, 0.46]
Constant	0.46*** (0.05)	0.56*** (0.08)	0.62*** (0.10)
	[0.36, 0.55]	[0.41, 0.72]	[0.42, 0.82]
Num.Obs.	208	194	194
R2	0.006	0.035	0.040
R2 Adj.	0.002	0.020	0.015
AIC	301.0	279.3	282.3
BIC	311.0	295.7	305.1
RMSE	0.49	0.48	0.48

Table S19: Clarity of consent: Is consent confusing?

	(1)	(2)	(3)
Pledge Type	0.11 (0.05)	0.10 (0.06)	−0.03 (0.12)
	[0.00, 0.21]	[−0.02, 0.21]	[−0.26, 0.20]
Gender		0.01 (0.06)	−0.04 (0.08)
		[−0.10, 0.12]	[−0.20, 0.11]
Freshman Status		0.05 (0.06)	0.00 (0.08)
		[−0.07, 0.17]	[−0.16, 0.16]
Pledge Type X Gender			0.11 (0.11)
			[−0.12, 0.33]
Pledge Type X Freshman Status			0.11 (0.12)
			[−0.13, 0.34]
Constant	−0.68*** (0.04)	−0.70*** (0.06)	−0.65*** (0.08)
	[−0.75, −0.60]	[−0.82, −0.58]	[−0.80, −0.50]
Num.Obs.	312	293	293
R2	0.012	0.013	0.018
R2 Adj.	0.009	0.003	0.001
AIC	435.0	415.8	418.3
BIC	446.2	434.2	444.1
RMSE	0.48	0.48	0.48

Table S20: Did you enjoy the party?

	(1)	(2)	(3)
Pledge Type	−0.04 (0.06)	−0.06 (0.06)	−0.04 (0.12)
	[−0.15, 0.08]	[−0.18, 0.05]	[−0.29, 0.20]
Gender		0.08 (0.06)	0.11 (0.08)
		[−0.04, 0.19]	[−0.06, 0.27]
Freshman Status		−0.04 (0.06)	−0.05 (0.09)
		[−0.16, 0.08]	[−0.22, 0.12]
Pledge Type X Gender			−0.06 (0.12)
			[−0.30, 0.17]
Pledge Type X Freshman Status			0.02 (0.13)
			[−0.23, 0.26]
Constant	0.57*** (0.04)	0.58*** (0.07)	0.57*** (0.08)
	[0.49, 0.65]	[0.45, 0.70]	[0.41, 0.73]
Num.Obs.	308	289	289
R2	0.001	0.011	0.012
R2 Adj.	−0.002	0.001	−0.005
AIC	449.3	422.4	426.1
BIC	460.5	440.8	451.8
RMSE	0.50	0.49	0.49

Table S21: Do you feel safe at this party?

	(1)	(2)	(3)
Pledge Type	−0.03 (0.05)	−0.03 (0.05)	−0.18 (0.12)
	[−0.13, 0.07]	[−0.13, 0.08]	[−0.42, 0.05]
Gender		0.15** (0.05)	0.11 (0.08)
		[0.05, 0.26]	[−0.04, 0.26]
Freshman Status		0.06 (0.06)	−0.01 (0.08)
		[−0.05, 0.17]	[−0.17, 0.14]
Pledge Type X Gender			0.09 (0.11)
			[−0.12, 0.31]
Pledge Type X Freshman Status			0.16 (0.12)
			[−0.07, 0.38]
Constant	0.72*** (0.04)	0.60*** (0.06)	0.67*** (0.08)
	[0.65, 0.79]	[0.48, 0.73]	[0.52, 0.82]
Num.Obs.	304	284	284
R2	0.001	0.030	0.038
R2 Adj.	−0.002	0.020	0.021
AIC	389.7	357.7	359.3
BIC	400.8	376.0	384.8
RMSE	0.45	0.45	0.44

Table S22: If you could, would you bring your little sister to this party?

	(1)	(2)	(3)
Pledge Type	0.00 (0.06)	−0.01 (0.06)	−0.11 (0.10)
	[−0.11, 0.11]	[−0.12, 0.10]	[−0.30, 0.09]
Gender		−0.02 (0.06)	0.01 (0.08)
		[−0.13, 0.09]	[−0.15, 0.17]
Freshman Status		0.28*** (0.05)	0.19* (0.08)
		[0.18, 0.39]	[0.03, 0.35]
Pledge Type X Gender			−0.06 (0.11)
			[−0.28, 0.16]
Pledge Type X Freshman Status			0.19 (0.11)
			[−0.02, 0.40]
Constant	0.38*** (0.04)	0.19** (0.06)	0.23** (0.07)
	[0.30, 0.45]	[0.08, 0.31]	[0.09, 0.38]
Num.Obs.	301	282	282
R2	0.000	0.077	0.088
R2 Adj.	−0.003	0.067	0.071
AIC	423.6	374.2	375.1
BIC	434.8	392.4	400.5
RMSE	0.48	0.46	0.46

Table S23: Is sexual assault a problem at [University]?

	(1)	(2)	(3)
Pledge Type	−0.47* (0.20)	−0.44* (0.20)	−0.33 (0.40)
	[−0.87, −0.08]	[−0.83, −0.05]	[−1.10, 0.45]
Gender		−0.87*** (0.20)	−0.81** (0.28)
		[−1.25, −0.48]	[−1.37, −0.26]
Freshman Status		0.35 (0.21)	0.39 (0.28)
		[−0.06, 0.77]	[−0.17, 0.94]
Pledge Type X Gender			−0.12 (0.39)
			[−0.90, 0.65]
Pledge Type X Freshman Status			−0.08 (0.43)
			[−0.91, 0.76]
Constant	0.49*** (0.14)	0.69*** (0.21)	0.64** (0.24)
	[0.21, 0.77]	[0.29, 1.10]	[0.16, 1.12]
Num.Obs.	305	286	286
R2	0.018	0.096	0.097
R2 Adj.	0.014	0.087	0.081
AIC	1216.8	1109.4	1113.3
BIC	1227.9	1127.7	1138.9
RMSE	1.76	1.65	1.65

4 Context Analysis

Table S24: Responsibility: How responsible are you for preventing sexual assault?

	(1)	(2)	(3)
Context	0.13 (0.07)	0.14* (0.07)	0.14 (0.07)
	[0.00, 0.26]	[0.00, 0.28]	[0.00, 0.28]
Pledge Type	0.17* (0.08)	0.21** (0.08)	0.21 (0.12)
	[0.02, 0.32]	[0.05, 0.36]	[−0.02, 0.44]
Context X Pledge Type	−0.25** (0.09)	−0.30** (0.10)	−0.30** (0.10)
	[−0.44, −0.07]	[−0.49, −0.11]	[−0.49, −0.11]
Gender		−0.02 (0.05)	−0.04 (0.07)
		[−0.12, 0.07]	[−0.17, 0.09]
Freshman Status		−0.13** (0.05)	−0.11 (0.07)
		[−0.22, −0.03]	[−0.24, 0.03]
Freshman Status X Pledge Type			−0.04 (0.10)
			[−0.23, 0.15]
Constant	0.46*** (0.06)	0.55*** (0.07)	0.55*** (0.08)
	[0.35, 0.57]	[0.41, 0.69]	[0.39, 0.72]
Num.Obs.	479	444	444
R2	0.015	0.035	0.036
R2 Adj.	0.009	0.024	0.020
AIC	693.2	637.9	641.5
BIC	714.0	666.5	678.3
RMSE	0.49	0.49	0.49

Table S25: Universality: Should everyone pledge?

	(1)	(2)	(3)
Context	0.06 (0.06)	0.04 (0.07)	0.04 (0.07)
	[−0.07, 0.18]	[−0.09, 0.17]	[−0.09, 0.17]
Pledge Type	0.08 (0.07)	0.07 (0.07)	0.16 (0.11)
	[−0.06, 0.22]	[−0.07, 0.22]	[−0.05, 0.37]
Context X Pledge Type	−0.26** (0.09)	−0.27** (0.09)	−0.28** (0.09)
	[−0.44, −0.08]	[−0.45, −0.09]	[−0.46, −0.09]
Gender		−0.14** (0.05)	−0.11 (0.06)
		[−0.23, −0.05]	[−0.23, 0.02]
Freshman Status		−0.07 (0.05)	−0.03 (0.07)
		[−0.16, 0.02]	[−0.16, 0.09]
Freshman Status X Pledge Type			−0.07 (0.09)
			[−0.25, 0.11]
Constant	0.62*** (0.05)	0.76*** (0.07)	0.72*** (0.08)
	[0.52, 0.72]	[0.63, 0.90]	[0.57, 0.88]
Num.Obs.	488	452	452
R2	0.029	0.061	0.063
R2 Adj.	0.023	0.050	0.048
AIC	677.4	617.2	620.3
BIC	698.4	646.0	657.3
RMSE	0.48	0.47	0.47

Table S26: Perceived norms: What % of your friends appreciate the pledge?

	(1)	(2)	(3)
Context	0.13 (0.08)	0.13 (0.09)	0.14 (0.09)
	[−0.03, 0.29]	[−0.04, 0.31]	[−0.04, 0.31]
Pledge Type	0.16 (0.09)	0.14 (0.10)	0.12 (0.15)
	[−0.03, 0.34]	[−0.06, 0.34]	[−0.18, 0.42]
Context X Pledge Type	−0.23* (0.12)	−0.24 (0.12)	−0.24 (0.12)
	[−0.46, −0.01]	[−0.48, 0.00]	[−0.48, 0.00]
Gender		−0.01 (0.06)	0.01 (0.08)
		[−0.12, 0.10]	[−0.15, 0.18]
Freshman Status		−0.09 (0.06)	−0.12 (0.09)
		[−0.22, 0.03]	[−0.29, 0.05]
Freshman Status X Pledge Type			0.06 (0.13)
			[−0.19, 0.31]
Constant	0.33*** (0.07)	0.40*** (0.09)	0.41*** (0.10)
	[0.19, 0.46]	[0.23, 0.57]	[0.21, 0.61]
Num.Obs.	315	295	295
R2	0.013	0.019	0.021
R2 Adj.	0.003	0.002	−0.003
AIC	454.2	427.5	431.1
BIC	472.9	453.3	464.3
RMSE	0.49	0.49	0.49

Table S27: Clarity of consent: Is consent confusing?

	(1)	(2)	(3)
Context	−0.14* (0.07)	−0.11 (0.07)	−0.12 (0.07)
	[−0.27, −0.01]	[−0.25, 0.02]	[−0.25, 0.02]
Pledge Type	−0.22** (0.07)	−0.25*** (0.07)	−0.40*** (0.11)
	[−0.36, −0.08]	[−0.39, −0.11]	[−0.62, −0.18]
Context X Pledge Type	0.33*** (0.09)	0.35*** (0.09)	0.36*** (0.09)
	[0.15, 0.50]	[0.17, 0.53]	[0.18, 0.54]
Gender		0.01 (0.05)	−0.05 (0.07)
		[−0.08, 0.10]	[−0.18, 0.08]
Freshman Status		0.00 (0.05)	−0.05 (0.07)
		[−0.09, 0.10]	[−0.18, 0.08]
Freshman Status X Pledge Type			0.12 (0.09)
			[−0.07, 0.30]
Constant	−0.54*** (0.05)	−0.56*** (0.07)	−0.49*** (0.08)
	[−0.65, −0.43]	[−0.70, −0.42]	[−0.65, −0.33]
Num.Obs.	487	452	452
R2	0.027	0.034	0.040
R2 Adj.	0.021	0.023	0.025
AIC	666.8	620.5	621.4
BIC	687.8	649.2	658.5
RMSE	0.47	0.47	0.47

Table S28: Did you enjoy the party?

	(1)	(2)	(3)
Context	0.25*** (0.06)	0.29*** (0.07)	0.30*** (0.07)
	[0.12, 0.38]	[0.16, 0.43]	[0.17, 0.43]
Pledge Type	−0.02 (0.07)	0.02 (0.07)	0.09 (0.12)
	[−0.16, 0.13]	[−0.13, 0.17]	[−0.14, 0.32]
Context X Pledge Type	−0.02 (0.09)	−0.08 (0.09)	−0.09 (0.09)
	[−0.20, 0.16]	[−0.27, 0.11]	[−0.28, 0.09]
Gender		0.05 (0.05)	0.10 (0.06)
		[−0.05, 0.14]	[−0.02, 0.23]
Freshman Status		−0.04 (0.05)	−0.04 (0.07)
		[−0.13, 0.06]	[−0.17, 0.09]
Freshman Status X Pledge Type			0.00 (0.10)
			[−0.19, 0.20]
Constant	0.32*** (0.05)	0.29*** (0.07)	0.26*** (0.08)
	[0.22, 0.42]	[0.16, 0.42]	[0.11, 0.41]
Num.Obs.	477	445	445
R2	0.053	0.063	0.066
R2 Adj.	0.047	0.052	0.051
AIC	674.3	629.4	631.8
BIC	695.2	658.0	668.6
RMSE	0.49	0.48	0.48

Table S29: Do you feel safe at this party?

	(1)	(2)	(3)
Context	−0.01 (0.06)	−0.01 (0.06)	−0.01 (0.06)
	[−0.13, 0.11]	[−0.13, 0.12]	[−0.13, 0.12]
Pledge Type	0.02 (0.07)	0.01 (0.07)	−0.02 (0.11)
	[−0.12, 0.15]	[−0.12, 0.15]	[−0.24, 0.19]
Context X Pledge Type	−0.04 (0.09)	−0.04 (0.09)	−0.03 (0.09)
	[−0.21, 0.12]	[−0.21, 0.14]	[−0.21, 0.14]
Gender		0.13** (0.04)	0.11 (0.06)
		[0.04, 0.22]	[−0.01, 0.23]
Freshman Status		0.01 (0.05)	0.00 (0.06)
		[−0.08, 0.10]	[−0.12, 0.12]
Freshman Status X Pledge Type			0.03 (0.09)
			[−0.15, 0.20]
Constant	0.73*** (0.05)	0.65*** (0.07)	0.67*** (0.08)
	[0.64, 0.83]	[0.52, 0.78]	[0.52, 0.82]
Num.Obs.	471	438	438
R2	0.002	0.022	0.022
R2 Adj.	−0.004	0.011	0.006
AIC	591.6	542.2	546.0
BIC	612.3	570.8	582.7
RMSE	0.45	0.44	0.44

Table S30: If you could, would you bring your little sister to this party?

	(1)	(2)	(3)
Context	0.07 (0.06)	0.03 (0.07)	0.03 (0.07)
	[−0.05, 0.20]	[−0.11, 0.16]	[−0.10, 0.17]
Pledge Type	0.07 (0.07)	0.05 (0.08)	0.08 (0.12)
	[−0.07, 0.22]	[−0.10, 0.21]	[−0.15, 0.31]
Context X Pledge Type	−0.07 (0.09)	−0.06 (0.10)	−0.07 (0.10)
	[−0.26, 0.11]	[−0.25, 0.13]	[−0.26, 0.12]
Gender		−0.04 (0.05)	0.01 (0.07)
		[−0.13, 0.06]	[−0.12, 0.14]
Freshman Status		0.20*** (0.05)	0.18** (0.06)
		[0.11, 0.29]	[0.05, 0.31]
Freshman Status X Pledge Type			0.05 (0.09)
			[−0.13, 0.23]
Constant	0.30*** (0.05)	0.22*** (0.07)	0.21** (0.08)
	[0.20, 0.41]	[0.09, 0.35]	[0.06, 0.36]
Num.Obs.	468	436	436
R2	0.003	0.044	0.047
R2 Adj.	−0.003	0.033	0.032
AIC	651.5	591.6	594.2
BIC	672.2	620.1	630.9
RMSE	0.48	0.47	0.47

5 All-Clubs Pledge

	No robust SEs	Robust SEs
22-day treatment period	−0.55 (0.37)	−0.55*** (0.14)
	[−1.27, 0.16]	[−0.84, −0.27]
Yearly 22-day period	0.14 (0.11)	0.14 (0.14)
	[−0.08, 0.35]	[−0.14, 0.41]
Treatment celebration night	4.37** (1.67)	4.37*** (0.65)
	[1.10, 7.64]	[3.10, 5.64]
Yearly celebration night	0.63 (0.48)	0.63 (0.65)
	[−0.31, 1.58]	[−0.64, 1.90]
Spring break	−0.48** (0.15)	−0.48*** (0.08)
	[−0.78, −0.18]	[−0.63, −0.33]
Summer break	−0.59*** (0.06)	−0.59*** (0.04)
	[−0.70, −0.48]	[−0.68, −0.51]
Fall break	−0.11 (0.15)	−0.11 (0.15)
	[−0.41, 0.19]	[−0.40, 0.18]
Thanksgiving break	−0.37 (0.21)	−0.37* (0.15)
	[−0.79, 0.05]	[−0.66, −0.08]
Winter break	−0.55*** (0.10)	−0.55*** (0.05)
	[−0.75, −0.34]	[−0.65, −0.45]
2007-2008 School Year	0.29* (0.12)	0.29*** (0.08)
	[0.06, 0.52]	[0.14, 0.44]
2008-2009 School Year	0.23 (0.12)	0.23*** (0.07)
	[0.00, 0.46]	[0.09, 0.36]
2009-2010 School Year	0.22 (0.12)	0.22** (0.08)
	[−0.01, 0.45]	[0.07, 0.37]
2010-2011 School Year	0.18 (0.12)	0.18* (0.08)
	[−0.05, 0.41]	[0.02, 0.34]
2011-2012 School Year	0.26* (0.12)	0.26** (0.09)
	[0.02, 0.49]	[0.08, 0.43]
2012-2013 School Year	0.23 (0.12)	0.23** (0.08)
	[0.00, 0.46]	[0.08, 0.38]
2013-2014 School Year	0.37** (0.12)	0.37** (0.12)
	[0.14, 0.61]	[0.13, 0.62]
2014-2015 School Year	0.33** (0.12)	0.33*** (0.09)
	[0.10, 0.56]	[0.16, 0.50]
2015-2016 School Year	0.41*** (0.12)	0.41** (0.13)
	[0.18, 0.64]	[0.14, 0.67]
2016-2017 School Year	0.44*** (0.12)	0.44*** (0.13)
	[0.21, 0.67]	[0.18, 0.70]
2018-2019 School Year	0.00 (0.12)	0.00 (0.06)
	[−0.23, 0.23]	[−0.11, 0.12]
Constant	0.42*** (0.09)	0.42*** (0.05)
	[0.25, 0.59]	[0.33, 0.51]
Num.Obs.	4375	4375
R2	0.041	0.041
R2 Adj.	0.037	0.037
AIC	16 336.6	16 336.6
BIC	16 477.1	16 477.1
Log.Lik.	−8146.325	
F	9.365	
RMSE	1.56	1.56
